# The Role of Galectin-1 in Cancer Progression, and Synthetic Multivalent Systems for the Study of Galectin-1

**DOI:** 10.3390/ijms17091566

**Published:** 2016-09-16

**Authors:** Jonathan M. Cousin, Mary J. Cloninger

**Affiliations:** Department of Chemistry and Biochemistry, Montana State University, Bozeman, MT 59717, USA; jonathanmcousin@gmail.com

**Keywords:** galectins, galectin-1, dendrimer, glycodendrimer, multivalent

## Abstract

This review discusses the role of galectin-1 in the tumor microenvironment. First, the structure and function of galectin-1 are discussed. Galectin-1, a member of the galectin family of lectins, is a functionally dimeric galactoside-binding protein. Although galectin-1 has both intracellular and extracellular functions, the defining carbohydrate-binding role occurs extracellularly. In this review, the extracellular roles of galectin-1 in cancer processes are discussed. In particular, the importance of multivalent interactions in galectin-1 mediated cellular processes is reviewed. Multivalent interactions involving galectin-1 in cellular adhesion, mobility and invasion, tumor-induced angiogenesis, and apoptosis are presented. Although the mechanisms of action of galectin-1 in these processes are still not well understood, the overexpression of galectin-1 in cancer progression indicates that the role of galectin-1 is significant. To conclude this review, synthetic frameworks that have been used to modulate galectin-1 processes are reviewed. Small molecule oligomers of carbohydrates, carbohydrate-functionalized pseudopolyrotaxanes, cyclodextrins, calixarenes, and glycodendrimers are presented. These synthetic multivalent systems serve as important tools for studying galectin-1 mediated cancer cellular functions.

## 1. Introduction

Multivalency, the binding of multiple ligands to multiple receptor binding sites [1,2], provides a platform to better understand cellular mechanisms that drive cancer metastasis. Multivalent protein–carbohydrate interactions mediate a myriad of malignant cellular processes, including cellular aggregation/tumor formation, metastasis, and angiogenesis [3,4,5]. These multivalent protein–carbohydrate interactions generally rely on multiple points of attachment to enhance the individual binding interaction between one carbohydrate and its receptor, which is typically weak [1,6,7].

Proteins that mediate multivalent malignant cellular activities are intriguing molecular targets [4]. Galectin-1, for example, is a multivalent carbohydrate binding protein that mediates the malignant cellular activities by cross-linking glycoproteins in the tumor microenvironment. Specifically, galectin-1 has been reported to be involved in multivalent mechanisms that cluster cell surface glycoproteins [8,9], cross-link receptors [10,11], and form lattices and larger aggregates [12,13,14].

Multivalent frameworks have proven to be powerful tools to modulate and study protein carbohydrate interactions. A variety of synthetic multivalent scaffolds including linear polymers [15,16,17], star [18,19,20] and hyperbranched [21,22,23] polymers, gold nanoparticles [24,25,26], dendrimers [27], proteins [28], beads [29] and surfaces [30,31,32,33] have been functionalized with carbohydrates and then applied to the study and the mediation of multivalent protein–carbohydrate interactions [34,35]. For example, these carbohydrate functionalized scaffolds have been used to study biological processes such as cellular aggregation/tumor formation [36], viral cell attachment [28,37], bacterial recognition [38], and signal transduction [39]. Many galectin-1 pathways are still not well understood, and there is a paucity of studies using multivalent frameworks to explore multivalent galectin-1 mechanisms. A better understanding of galectin-1 mechanisms can advance the overall understanding of malignant cellular activities and give insight into the rational design of multivalent therapeutics.

This review discusses the role of galectin-1 in the tumor microenvironment. First, the structure of galectin-1 is discussed. Multivalent interactions involving galectin-1 in cellular adhesion, mobility and invasion, tumor-induced angiogenesis, and apoptosis are presented. To conclude this review, synthetic glycosylated frameworks that have been used to study and to modulate galectin-1 processes are reviewed. The synthetic multivalent frameworks have served as important tools to establish the role of multivalent binding interactions for the galectin-1 mediated advancement of cancer processes.

## 2. Structure of Galectin-1

### 2.1. The Galectin Family of Lectins

Galectin-1 is one of 15 members of the β-galactoside binding family of proteins called the galectins, which share a conserved amino acid sequence in the carbohydrate recognition domain (CRD) [40,41]. The galectin family can be subdivided into three groups based on the structure of the protein: (i) monovalent galectins containing one CRD that are capable of homodimerizing to become functionally bivalent; (ii) bivalent galectins with two non-identical CRDs connected by a peptide linker; and (iii) chimeric galectins with one CRD and a unique N-terminus [42,43]. Galectin-1, -2, -5, -7, -10, -11, -13, -14, and -15 have one CRD and are capable of forming homodimers. Those with two dissimilar CRDs connected by a short linker peptide include galectin-4, -6, -8, -9, and -12. Galectin-3 is the only chimera-type; this protein consists of a C-terminal CRD fused to a non-lectin N-terminal domain composed of tandem repeats of short amino-acid stretches that participates in oligomerization [42,44]. Glycan-binding specificity, protein valency, and cross-linking properties of individual galectins differentiate their biological responses [11,42,45,46,47,48]. Of the 15 members of the lectin family, galectin-1 and galectin-3 appear to be the major players in cancer biology and, therefore, have stimulated significant research interest [48,49,50]. However, less is known about galectin-1 pathways compared to galectin-3.

### 2.2. Galectin-1

**Figure 1:**
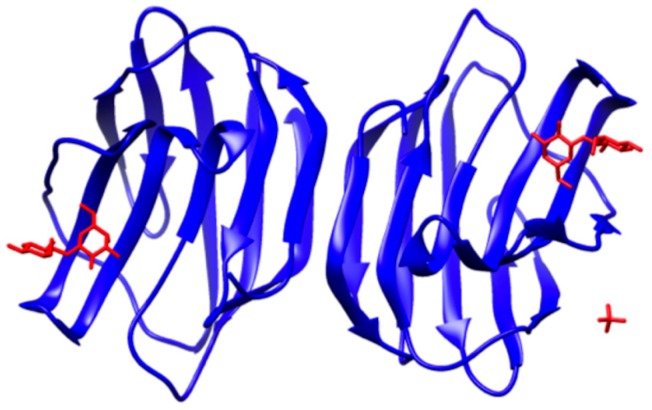
Dimeric structure of galectin-1. Galectin-1 (**blue**) with lactose (**red**) bound in the apposing carbohydrate recognition domains. Reproduced with permission from Reference [52].

Galectin-1 is a homodimeric protein composed of 14.5 kDa subunits [40]; the dimer is maintained by hydrophobic interactions at the monomeric interface and by the well-defined hydrophobic core (Figure 1) [51]. As shown in Figure 1, the monomeric units are anchored such that the two CRDs are located on opposing ends of the quaternary structure at a distance of approximately 5 nm [13]. Each CRD is able to accommodate a tetrasaccharide [44]. The apposing CRDs initiate cellular recognition and signal transduction events by binding appropriately glycosylated ligands [44]. The importance of valency and of the interplay between monovalent and multivalent forms to the function of galectin-1 have not been made clear. As presented in the final section of this review, multivalent materials represent an important tool for advancement of the understanding of how galectin-1 structure impacts function.

### 2.3. Galectin-1 Synthesis and Secretion

As is characteristic of cytoplasmic proteins, galectin-1 is synthesized on cytosolic ribosomes and possesses the archetypal acetylated N-termini and lack of signal peptides [44]. From the cytosol, galectin-1 can be targeted to cell nuclei, translocated to the intracellular side of cell membranes, or secreted [44,51]. Secretion occurs through an unorthodox mechanism that involves direct translocation across the plasma membrane, bypassing the classical vesicle-mediated endoplasmic reticulum/Golgi pathway for exocytosis [42,51]. This novel export machinery has been proposed to use β-galactoside-containing surface molecules as export receptors for intracellular galectin-1 [42,51]. By specifically targeting the β-galactoside binding site motif, this export machinery would provide a quality control mechanism to recognize only properly folded galectin-1 [51,53]. Although galectin-1 lacks identifiable secretion signal sequences, it is found on the extracellular side of cell membranes and in the extracellular matrix (ECM) of various normal and neoplastic tissues [44,51].

## 3. Overview of Galectin-1 in Cancer

The amount of extracellular of galectin-1 is altered in a variety of cancer cell types [54,55,56], including melanoma [57,58], ovarian [59,60], lung [61], prostate [62,63], bladder [64], thyroid [65,66], pancreatic [67], head-neck [68], cervical [69], uterine [70], and colorectal cancers [71,72]. In addition, galectin-1 is often overexpressed in the stroma surrounding tumor cells [73,74].

The increased expression of galectin-1 has been correlated with a variety of processes in cancer progression, including the cellular aggregation/tumor formation, metastatic spread of cancer, angiogenesis, and apoptosis [5,48,51,75,76]. A brief summary of galectin-1 binding partners, cellular location, and associated activities in cancer is provided in Table 1. More detail regarding the binding partners and associated biological activities of galectin-1 are provided in recent reviews [5,42,47,48,51,77].

### 3.1. Intracellular Galectin-1

Intracellular galectin-1 acts as a scaffold protein for intracellular signaling pathways in a carbohydrate independent manner. Relevant to cancer cell biology, intracellular galectin-1 participates in protein–protein interactions with H-Ras [78], protocadherin-24 [79], and Gemin4 [80] in a carbohydrate-independent manner [48,51]. These proteins are structurally unrelated and do not share any common domains or motifs [51].

### 3.2. Extracellular Galectin-1

Extracellular galectin-1 participates in the defining β-galactoside binding activity [42,57,63,82,87,88]. For example, the metastatic spread of cancer occurs in part through interactions between galectin-1 and glycoproteins in the ECM, such as laminin and fibronectin [51]. The binding of cell-surface glycoproteins, such 90K/Mac-2BP and Mucin 1, by galectin-1 mediates cellular aggregation/tumor formation [51]. Lactose-functionalized dendrimers were shown to nucleate the formation of galectin-1/glycodendrimer aggregates of about 500 nm diameter that were remarkably homogeneous. When added to cell in vitro, these galectin-1/glycodendrimer aggregates provided an alternative to ECM binding that altered cell surface processes [89].

Galectin-1 mechanisms in cellular aggregation/tumor formation, migration and invasion, tumor-induced angiogenesis, and apoptosis of activated T cells are discussed in detail in the following sections of this review. In particular, multivalent functions of galectin-1 are highlighted.

## 4. Multivalent Mechanisms of Action of Galectin-1 in Cancer

This section will address multivalent galectin-1 mediated mechanisms in cellular aggregation, adhesion to the ECM, migration and invasion, angiogenesis, and apoptosis.

### 4.1. Homotypic Cellular Aggregation

Galectin-1 has been reported to mediate homotypic aggregation of cancer cells through multivalent interactions with cell-surface glycoconjugates in a variety of cell types [48,49]. Tinari et al. demonstrated that galectin-1 induces homotypic aggregation in human melanoma cells (A375) [57]. Galectin-1 was observed to bivalently bind the cell surface glycoprotein 90K/Mac-2BP on adjacent cells to promote aggregation [57].

Mucin 1 is another putative cancer cell-surface receptor of galectin-1 [51,90]. Mucin 1 is a large transmembrane protein that is overexpressed and aberrantly glycosylated with the Thomsen–Friedenreich (TF) antigen (a Galβ1-3GalNAc disaccharide) on many cancer cells [91]. Galectin-1 binding to the TF antigen was reported by Jeschke et al. [58]. The TF antigen was conjugated on a polyacrylamide framework (TF-PAA), and galectin-1 binding to TF-PAA was monitored by following cellular aggregation using a chorionic carcinoma cell line (BeWo) known to express Mucin 1 [58]. TF-PAA was observed to strongly diminish the interactions that drive cellular aggregation in the BeWo cell line, indicating that the binding of galectin-1 to the TF antigen expressed on Mucin 1 mediated cellular aggregation [81].

Providing further support for the galectin-1 TF antigen interaction as a significant pathway in cancer cell aggregation, Glinsky et al. observed the interaction of galectin-1 with the TF-antigen on MDA-MB-435 breast cancer cells [92]. Using confocal microscopy, galectin-1 accumulation was observed at the interface between MDA-MB-435 cells [92]. Aggregation was inhibited by the TF-antigen specific peptide P-30, indicating the galectin-1 binding to the TF-antigen mediated homotypic aggregation [92].

**Figure 2:**
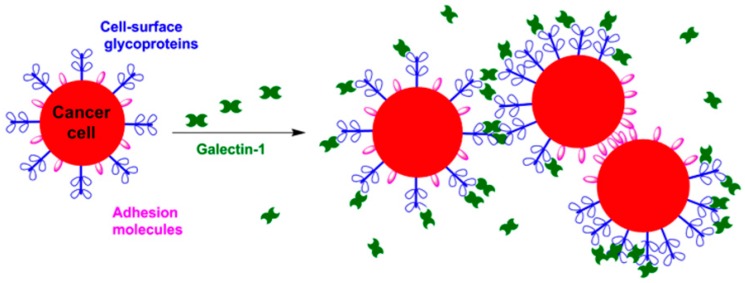
Galectin-1 mediates homotypic aggregation of cancer cells through multivalent interactions with cell-surface glycoproteins on adjacent cells and through reorganization of the cell surface, which exposes adhesion molecules.

These studies indicate that multivalent interactions of galectin-1 with cell-surface glycoconjugates (e.g., Mucin 1) mediate aggregation through cellular cross-linking (Figure 2) [88]. Multivalent binding of TF antigen by galectins has been reported to cluster Mucin 1 on the cell surface, which facilitates cellular aggregation by exposing adhesion molecules [90,93]. Although the mechanism of galectin-1–Mucin 1 mediated cellular aggregation is not fully resolved, it is likely similar to that of galectin-3. Galectin-3 mediates cellular aggregation by binding the TF antigen expressed on Mucin 1 [90]. Galectin-3 binding to Mucin 1 in the human colon cancer cell line HT-29 was monitored by confocal microscopy, which revealed that the interaction of galectin-3 with Mucin 1 polarized the cell surface [90]. Mucin 1 becomes clustered on the surface of the cell, which exposes smaller adhesion molecules previously concealed by the large transmembrane protein [90]. Exposed adhesion molecules interact with adhesion molecules on neighboring cells to aggregate cells. Because both galectin-1 and galectin-3 are known to bind to the TF antigen on Mucin 1 [58], it is therefore likely that galectin-1 mediates aggregates through a similar a mechanistic pathway. Thus, it is likely that galectin-1 multivalently mediates cellular aggregation by cross-linking adjacent cells and exposing adhesion molecules through the clustering of Mucin 1 (Figure 2).

Inhibition of cancer cellular aggregation in vitro was demonstrated using lactose-functionalized dendrimers. Galectin-1 was bound by the glycodendrimers, which disabled the process shown in Figure 2. Glycodendrimers nucleated the formation of glycodendrimer/galectin-1 aggregates such that galectin-1 no longer effectively cross-linked the cancer cells. Fluorescence labeling studies also indicated that multivalent binding of galectin-1 to reorganize cell surface Mucin 1 was also disrupted in the presence of glycodendrimers [89]. This study strongly supports the hypothesis that galectin-1 uses multivalent interactions to aggregate cancer cells.

### 4.2. Cellular Adhesion to the Extracellular Matrix (ECM)

Galectin-1 has been observed to arbitrate the adhesion of cancer cells to the ECM [63,83]. Laminin, fibronectin, and other glycoproteins presented in the basement membrane provide the necessary epitopes for galectin-1 cell–ECM cross-linking [77,94]. A comprehensive list of basement membrane proteins and associated biological activities is provided in the referenced review [77].

**Figure 3:**
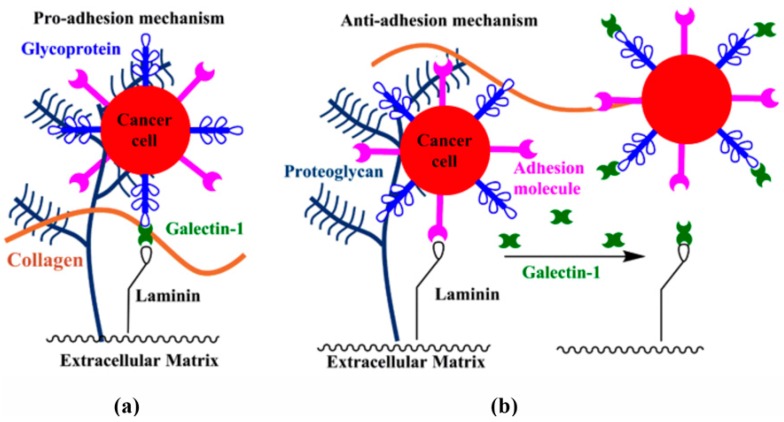
Biphasic arbitration of cell–extracellular matrix (ECM) interactions by galectin-1: (**a**) galectin-1 mediated cross-linking of cell-surface glycoconjugates and ECM glycoproteins promotes adhesion; and (**b**) competitive binding to ECM glycoproteins by galectin-1 inhibits adhesion and promotes dissemination of tumor cells.

Galectin-1 has been observed to biphasically arbitrate cell–ECM interactions. Dose-dependent binding of galectin-1 to laminin and fibronectin in cell–ECM adhesion has been reported in melanoma [82] and ovarian [83] cancer cell lines. As shown in Figure 3a, the pro-adhesive mechanism proceeds through galectin-1 mediated cross-linking of ECM proteins and cell-surface glycoconjugates. These studies suggest that one activity of galectin-1 is to modulate cancer cell adhesion during metastasis. Conversely, negative regulation of cell–ECM adhesion, in which galectin-1 inhibits cell–ECM interactions by competitively binding matrix glycoproteins or cell-surface glycoconjugates, has also been well documented (Figure 3b). In studies by the Barondes’ group, differentiating C2C12 mouse myoblasts were adhered to a laminin-immobilized surface [95]. Exogenous galectin-1 was observed to inhibit adhesion to and migration on the lamimin-coated plates [95]. Similar anti-adhesive properties have been observed with other cell types in the presence of exogenous galectin-1 [96,97,98]. These studies suggest another role for galectin-1—the modulation of tumor cell detachment, which allows tumor cells to travel to a secondary site.

Taken together, these studies demonstrate that galectin-1 binding to ECM glycoproteins arbitrates both cancer cell adhesion and detachment. Factors that may account for the biphasic activity include: (i) expression of cell-type dependent glycoconjugate ligands for galectin-1; (ii) expression of cell-type dependent receptors for ECM proteins that influence galectin-1 binding; (iii) co-expression of multiple galectins exhibiting antagonistic properties; and (iv) changes in expression levels and/or oligomerization of galectin-1 [77]. Although shown schematically in Figure 3 as a dimer, changes in the effective valency of galectin-1 could significantly impact ECM adhesion.

### 4.3. Metastasis: Cancer Cell Migration and Invasion

The hematogenous dissemination of cancer cells is essential to metastasis. During the metastatic spread of cancer, malignant cells detach from a primary site and migrate to and invade a secondary site [94]. Galectin-1 expression has been identified as a signature of cellular invasiveness of mammary carcinoma cell [99]. Furthermore, elevated levels of galectin-1 have been measured in the tissue at the invasion front of glioblastoma tumors [100,101,102] and oral squamous cell carcinomas [103]. A comprehensive list of cancer cell types in which galectin-1 has been observed to mediate migration and invasion is provided in a recent review [77].

**Figure 4:**
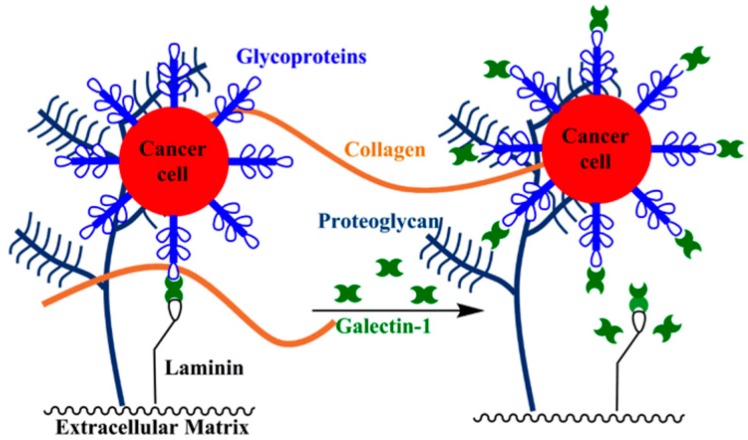
Galectin-1 competitively binds receptors involved in cell–ECM adhesion to promote migration and invasion.

Galectin-1 is involved in multiple metastatic processes: (1) adhesion of tumor cells to the ECM [77,94]; (2) binding of ECM glycoproteins [5,77]; and (3) enhancing proteolytic enzyme pathways [104,105]. Initially, galectin-1 multivalently mediates tumor cell–ECM adhesion at the primary site by cross-linking cell surface glycoproteins, such as integrins [94], and glycosylated proteins in the ECM [106], such as laminin and fibronectin [49]. Galectin-1 then directly mediates migration and invasion by competitively binding receptors involved in cell–ECM interactions, which allows cancer cells to detach from the primary site (Figure 4) [5,77]. Pursuant to this mechanism, a correlation likely exists between a tumor cell’s transformation to a migratory phenotype and galectin-1 expression levels: the amount of extracellular galectin-1 is likely increased in order to competitively bind glycoconjugates involved in cell–ECM cross-linking interactions. Endothelial cell migration has been impaired by interfering with endogenous galectin-1 expression by, for example, siRNAs or antisense nucleotides, which supports the direct involvement of galectin-1 binding in tumor cell migration [61,107,108]. Once the tumor cell migrates to and invades a secondary site, galectin-1 mediates adhesion by cross-linking tumor cells with the ECM.

Extracellular galectin-1 has also been observed to stimulate up-regulation and secretion of proteolytic enzymes, such as matrix metalloproteinases (MMP), that degrade ECM glycoproteins [104,109]. Activation of the signal transduction pathway that triggers secretion of proteolytic enzymes most likely occurs through galectin-1 mediated multivalent clustering of cell-surface receptors.

### 4.4. Tumor-Induced Angiogenesis

This section will discuss galectin-1 mediation of proangiogenic pathways. Angiogenesis is a complex processes and has been reviewed in great detail [110,111]. Here, a brief overview of angiogenesis will be provided followed by a discussion of galectin-1 mechanisms in angiogenesis.

**Figure 5:**
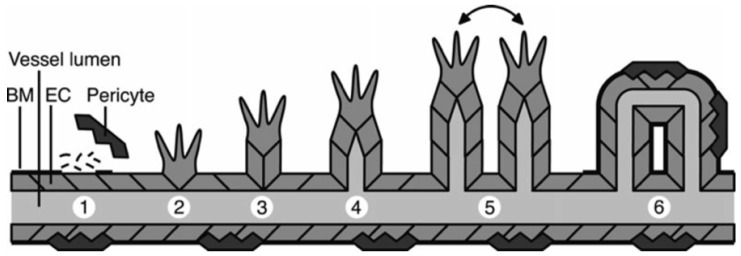
Angiogenesis. Illustration of the angiogenesis cascade that involves: (1) pericyte detachment and basal membrane degradation in response to endothelial cell activation; (2) migration of endothelial tip cells in the direction of the growth factor gradient; (3) provision of support of endothelial tip cells by the underlying stalk cells; (4) continuation of this process to form luminized vessel sprouts; (5) fusion of sprouts; and (6) formation of a functional vessel which is further stabilized by deposition of a basal membrane and attraction of pericytes for structural support [3]. Figure reproduced with permission from Reference [3]. BM, basal membrane; EC, endothelial cell.

Angiogenesis is the growth of new blood vessels out of preexisting capillaries. Blood vessels are fundamentally composed of endothelial cells, and once activated, these cells can extend the vascular network by interconnecting to form tubes that direct and maintain blood flow [110]. Tubule formation involves matrix degradation, proliferation, migration, tube formation, and matrix deposition and is depicted in Figure 5 [3].

Angiogenesis occurs naturally during ovulation and wound healing [110]. Tumor-induced angiogenesis, on the other hand, is a pathologic condition in which tumor cells secrete growth factors, such as vascular endothelial growth factors (VEGFs), to promote the growth of new blood vessels [112,113]. These growth factors activate quiescent vasculature in host tissue to stimulate the growth of new capillaries [110]. The growth of new capillaries is an indispensable process of metastasis–angiogenesis must occur for tumors to grow beyond a critical size of a few square millimeters [110,114]. New blood vessels generated from existing vasculature provide tumors with the necessary blood supply, oxygen, and nutrients for proliferation. Thus, the neovascular system provides a critical interface between cancer cells and host tissue.

During angiogenesis, endothelial cells are involved in different processes to form the neovasculature, and galectin-1 binding of glycoconjugates mediates many of these processes. Elevated levels of galectin-1 expression have been observed in the vasculature of many tumors, including prostate [107,115], lung [116], colon [117], and oral [61]. While galectin-1 has been observed to enhance tumor-induced angiogenic processes [113,118,119], the mechanisms of action have not been fully resolved.

It is likely that the glycosylation patterns of cell surface receptors (e.g., vascular endothelial growth factor receptors (VEGFRs) and Neuropilin 1 (NRP-1)) discriminates galectin-1 binding. Croci et al. observed that glycan specific binding of galectin-1 to VEGFR-2 promoted endothelial cell, EC, signaling and preserved the angiogenic phenotype in the absence of the putative binding partner, VEGF-A [119]. Glycome remodeling of the EC surface facilitated binding of galectin-1 to *N-*glycans expressed on VEGFR-2 in anti-VEGF refractory tumors but inhibited galectin-1 binding in anti-VEGF sensitive tumors, which explains the proliferation of certain tumor types during anti-VEGF treatment [119]. In contrast to anti-VEGF-sensitive tumors, which display high levels of α2-6-linked sialic acid glycans that inhibit galectin-1 binding, anti-VEGF refractory tumors exhibit vasculature glycosylation patterns (e.g., β1-6GlcNAc) that facilitate interactions with galectin-1 and an increase in galectin-1 expression [119].

**Figure 6:**
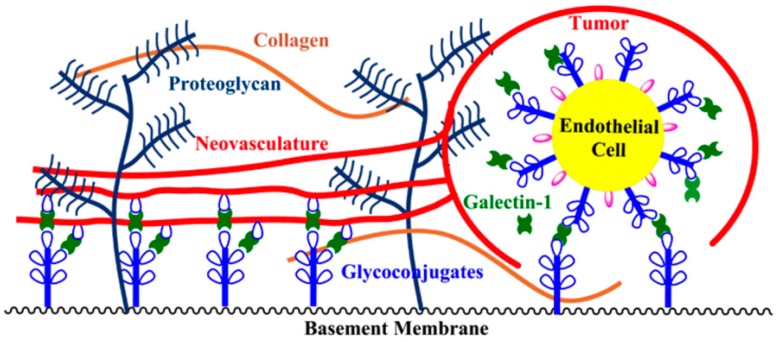
Provision of structural support for neovasculature by galectin-1.

Tumor vascularization has been correlated with elevated levels of galectin-1 in the endothelium [9,117,118]. In addition to activating endothelial cells, it has been proposed that galectin-1 plays a critical role in cellular adhesion and migration, functions that are important for angiogenesis [86]. Vascular endothelial cells have highly glycosylated cell-surfaces [50], and the ECM in the tumor stroma contains significant levels of known galectin-1 receptors laminin and fibronectin [50]. Activated endothelial cells, after invading the stroma, adhere to the ECM to form a lumen of a new capillary tube [110]. Capillary tubes then coalesce into loops, forming the new vasculature paramount for tumor growth and metastasis [110]. Galectin-1 likely acts as a scaffold for vessel growth and vascular network formation by establishing physical connections between vascular endothelial cells and the ECM in the tumor microenvironment, which provide the necessary physical support for neovasculature [27,107,108]. One way that this could be envisioned is shown in Figure 6. For example, the addition of galectin-1 to endothelial cells (EAhy926 cells and human umbilical vein endothelial cells (HUVECs)) causes an enhancement in microtubule formation, which is the in vitro model for angiogenesis [9,120].

In galectin-1 knockout experiments using mice, impedance of tumor growth due to inadequate angiogenesis has been observed [107,108]. Furthermore, in vivo inhibition studies of galectin-1 activity in the chick chorioallantoic membrane revealed tortuous and irregular neovessel growth in the absence of galectin-1, indicating defective vascular guidance [107]. Interestingly, galectin-1 inhibition decreased neovascularization in a manner similar to anginex, a galectin-1 specific angiogenesis inhibitor [107].

These studies indicate that galectin-1 augments key angiogenic pathways by mediating the activation of endothelial cells and the formation of a neovasculature network through multivalent carbohydrate binding [61,107,108,113,117]. Because galectin-1 binding of glycoconjugates mediates many key processes in angiogenesis, the critical roles of galectin-1 in the angiogenesis cascade remain an area of active, important investigation.

### 4.5. Galectin-1-Induced T Cell Apoptosis

The poor prognosis associated with elevated galectin-1 expression is related to tumor evasion of the immune response [51]. Tumors evade the immune response by secreting galectin-1, which triggers apoptosis of infiltrating T cells. This section will discuss the mechanism of galectin-1-induced apoptosis of activated T cells that allows tumors to evade the immune response.

**Figure 7:**
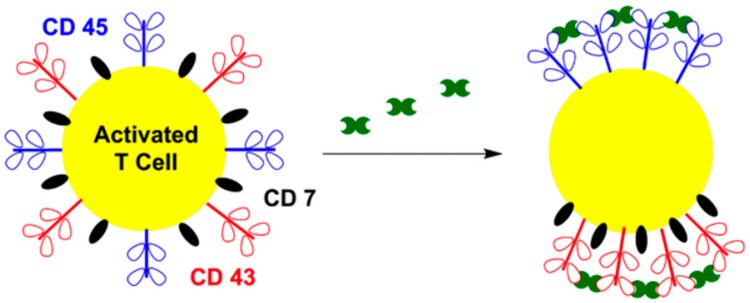
Galectin-1 mediated T cell apoptosis. Galectin-1 induces segregation and clustering of CD45 in distinct microdomains from CD43/CD7 complexes. Green motif, galectin-1.

Galectin-1 regulates apoptotic signaling pathways through colocalization of receptors into signaling complexes [11,12,39,121]. CD45, CD43, and CD7 have been identified as specific apoptotic-related receptors for galectin-1 [85]. As shown in Figure 7, galectin-1 cross-linking induces segregation and clustering of these receptors into discrete membrane microdomains, which are capable of transducing the cell death signal [85]. Pace et al. performed immunofluorescent localization studies using confocal microscopy to monitor the localization of the receptors before and after galectin-1 treatment [85]. Prior to galectin-1 treatment, receptors CD45 and CD43 were randomly distributed across the cell surface [85]. Galectin-1 binding resulted in the redistribution of these receptors and localization of CD45 in apoptotic membrane blebs [85]. Interestingly, CD43 was observed to segregate from CD45 and to colocalize with CD7 in distinct blebs on the cell-surface [10,85]. These results suggest that CD43 may act as a galectin-1 concentrating agent, facilitating subsequent interactions between galectin-1 and CD7 [10]. Furthermore, only dimeric galectin-1 activates the apoptotic signaling pathway that triggers the death of activated T cells, which indicates that bivalent cross-linking of cell-surface receptors mediates transduction of apoptotic signals [86].

Localization of apoptotic receptors into homogenous signaling complexes is critical to T cell apoptosis: the signaling pathway that transmits the cell death signal is activated upon galectin-1 mediated segregation and localization [10]. A remarkable characteristic of the galectin-1 apoptosis mechanism is the ability of the protein to discriminate among and homogenously cluster cell-surface receptors via selective cross-linking. In addition to the β-galactoside specificity provided by the hydrogen bonding amino acids in the CRD, the extended binding pocket of galectin-1 permits discrimination among receptors in terms of the galactosides composition of the receptor [45].

A distinct mechanism for galectin-1-mediated immunosuppression was proposed by Rubinstein et al., whereby tumor secreted galectin-1 impairs T cell effector functions involved in eradicating cancer cells from a host [122]. Effector impairment was suggested to occur either by activation of T cell apoptotic signaling pathways or by elevation of the activation threshold of naive T cells [122]. In light of the findings by Pace et al. discussed above, it is likely that galectin-1-mediated T cell receptor clustering activates T cell apoptotic signaling pathways involved in immunosuppression.

In support of the role of galectin-1 in tumor immunosuppression, in vivo blockage of galectin-1 expression in tumor cells of syngeneic mice promoted tumor rejection and stimulated tumor-specific T cell-mediated responses [122]. Upon subsequent treatment with galectin-1 sufficient tumors, the syngeneic mice exhibited an enhanced tumor immune response by resisting tumor challenge [122]. Together, these results demonstrate the importance of galectin-1 in tumor immunosuppression.

## 5. Synthetic Multivalent Systems for Binding of Galectin-1

From the above discussion of galectin-1 mediated cellular mechanisms in cancer, it should be understood that galectin-1 interacts multivalently with glycosylated receptors throughout the metastatic progression of cancer. Because a single interaction between a carbohydrate and its receptor (e.g., protein) is attenuated [7], nature presents multiple copies of receptors to enhance individual binding interactions and to elicit a biological response [1,2]. To better understand multivalent protein–carbohydrate interactions in cancer, synthetic multivalent frameworks can be applied to modulate native multivalent cellular mechanisms [123]. Multivalent nanoparticles functionalized with peptide and small interfering RNA (siRNA) ligands for galectin-1 have recently been reviewed [124]. Multivalent systems can be used for targeted drug delivery, imaging, and to discern and mediate multivalent biological processes. An understanding of the impact that valency has on galectin-1 mediated cancer processes is important for advancement of our mechanistic understanding of the impacts of galectin-1 on cancer progression. Although homotypic cellular aggregation, cellular adhesion to the ECM, metastasis, angiogenesis, and apoptosis are highlighted here, additional processes in which multivalent binding by galectin-1 is involved in cancer progression are likely. In this section, carbohydrate-functionalized synthetic multivalent systems that have been reported in studies with galectin-1 are described.

### 5.1. Dimers and Small Clusters of Carbohydrates

**Figure 8:**
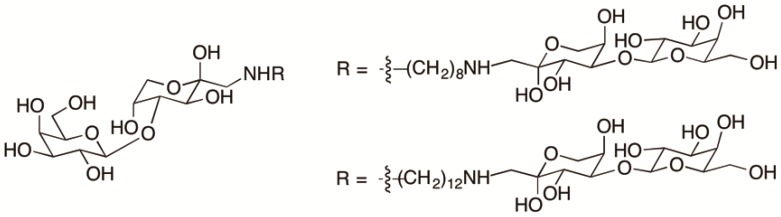
Lactulose amine dimers to target galectin-1.

Rabinovich et al. synthesized lactulose amine dimers to target galectins (Figure 8) [125]. The lactulose amine dimers inhibited binding of galectin-1 to the protein 90K in solid-phase assays. In addition, the dimers demonstrated regulatory effects in galectin-1 mediated tumor-cell apoptosis, homotypic cellular aggregation, and angiogenesis. For example, the dimer connected by eight carbons inhibited homotypic cellular aggregation and angiogenesis whereas the 12 carbon chain dimer did not. However, apoptosis was enhanced when in the presence of either dimer [125].

The length, rigidity, and functionality of the linker group have repeatedly been shown to have important consequences for multivalent binding. Linkers that are too short, too long, or rigidly oriented in such a way that multivalent interactions become strained reduce the likelihood that multivalent binding will be impactful, since unfavorable enthalpic contributions to binding will be introduced. Linkers that are too hydrophobic or introduce unfavorable noncovalent interactions outside the binding pocket can also negate any gains that would be realized via multivalency [1,126,127,128,129]. Thus, the length of the linker in this case, although not discussed in the cited article, is likely impacting either the ability of the lactose ligands to bind efficiently to their receptors or the ability of the linker portion to avoid unfavorable stereoelectronic interactions outside the receptor binding site. 

In another interesting study of the impact of linkers on binding, isothermal titration calorimetry (ITC) and hemagglutination studies were performed on a series of short dimers of *N*-acetyllactosamine by Ahmad et al. [130]. Linkers comprised of two, three, or four methylene units as well as a galactoside core were used to link *N*-acetyllactosaminosides. In this case, agglutination and ITC values were comparable for all of the compounds that were studied [130]. When rigid linkers were used to create dimers and tetramers of lactosides, no significant multivalency effect was observed in an enzyme linked immunosorbent assay (ELISA) type inhibition assay for binding to galectin-1, but differences in binding between galectin-1 and galectin-3 were observed [126]. Longer linkers may be required for significant changes in binding to be detected with galectin-1 because the carbohydrate binding sites are pointing in opposite directions. Cross-linking and matrix formation, which is difficult to quantify with dimers and small clusters using many traditional binding experiments, may be more impactful for function with galectin-1 than with some of the other lectins that are routinely used for the study of protein–carbohydrate interactions. An example in which dimers, trimers, and tetramers of lactose were actually less effective than lactose itself at inhibiting surface binding of galectin-1 to an immobilized target was provided by Andre et al. [131]. This example demonstrates the capability of the linker to negatively impact associations. On the other hand, incorporation of a carbamate in the linker for lactose dimers and trimers improved binding affinities and selectivities for galectin-1 relative to linkers containing amides [132].

Taken together, these studies of small clusters of carbohydrates binding to galectin-1 highlight the importance of the nature of the linker units. The studies reported here also draw attention to the importance of crosslinking, i.e., binding across multiple galectin-1 dimers rather than binding to both of the carbohydrate binding sites on one galectin-1 homodimer.

### 5.2. Self-Assembled Pseudopolyrotaxanes

Rotaxanes are mechanically interlocked molecules in which one molecule threads through another, as with a string passing through a bead on a necklace. Polyrotaxanes have multiple “bead” molecules threaded onto one macromolecule or polymer “thread”. Pseudopolyrotaxanes retain the concept of multiple “bead” molecules along a “thread”, but no endgroups are present on the “thread” to prevent movement of the “bead” molecule on and off the thread [133,134]. Thus, the pseudopolyrotaxanes represent the most versatile and most dynamic members of this family of molecules.

**Figure 9:**
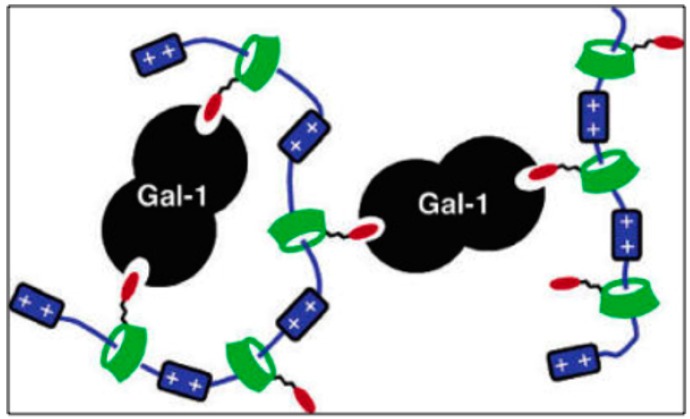
Self-Assembled Pseudopolyrotaxanes, statistical binding mechanism for Galectin-1. Reprinted with permission from [136]. Red is the carbohydrate, blue is a bipyridinium segment, and green is the cyclodextrin.

Belitsky et al. designed self-assembled pseudopolyrotaxanes as flexible and adaptable multivalent ligands for galectin-1 (Figure 9) [135]. Lactoside-displaying cyclodextrin units were non-covalently conjugated on a polymeric polyviologen backbone. Rotational and translational freedom along the polymeric backbone provides a mobile display of lactosides. Agglutination assays revealed that galectin-1 interacted with flexible multivalent ligands with higher affinity than compared to less dynamic ligand displays. Multivalent inhibition enhancement was attributed to multivalent lactosides successively binding the galectin-1 CRD, which effectively decreased *k*_off_ [135]. As depicted in Figure 9, this pseudopolyrotaxane system is much more likely to effectively cross-link galectin-1 lectins than small glycoclusters [136].

### 5.3. Carbohydrate-Functionalized Cyclodextrins and Calixarenes

Calixarenes are cyclic oligomers of phenols with formaldehyde. Calixarenes were functionalized with two to eight lactoside or galactoside groups on their upper rim by Andre, Ungaro and co-workers. Because of their relatively rigid structure, the carbohydrate-functionalized calixarenes were used to determine the impact that the orientation of ligands (in addition to their density) would have on binding specificity and selectivity with galectins. Functionalization of the lower rim of the calixarene (opposite the sugar ligands) with alkyl groups locked the calixarenes into fixed conformations that were used in binding assays. In solid phase inhibition assays with asialofetuin-functionalized surfaces, differences in the calixarene conformations caused differences in binding selectivity amongst the galectins [137].

β-Cyclodextrins with galactose, lactose, and *N*-acetyllactosamine functionalizations were used in inhibition binding assays with surface-presented carbohydrates and galectin-1, galectin-7, or galectin-3. Galectin-3 was found to bind more effectively to the functionalized cyclodextrin than galectin-1 and galectin-7 [138].

Binding studies using carbohydrate functionalized calixarenes and cyclodextrins demonstrate the importance that the alignment of the carbohydrates on a multivalent framework plays in creating selective binding partners for galectins.

### 5.4. Glycodendrimers

Dendrimers are a powerful framework to investigate multivalent protein–carbohydrate interactions and to mediate associated biological activities. The polymeric framework is symmetrical and highly branched. Dendrimers have sizes that allow for biomimicry of receptors [139] and contain a variable number of end groups for synthetic modifications [140]. These characteristics contribute to the utility of dendrimers in biological studies and applications [141], such as DNA- and drug-delivery [142], tissue repair [143], and cellular imaging [144].

Andre and co-workers developed small dendrimers bearing two, four, or eight lactosides with a 3,5-di(2-aminoethoxy)benzoic acid branching unit. These dendrimers were used as inhibitors for galectin-1 binding to glycoprotein-functionalized surface including laminin, and differences in the effectiveness of inhibition were observed for galectin-1 relative to galectin-3 and galectin-7. The best inhibition was observed for galectin-1 [145].

For poly(amidoamine) (PAMAM) dendrimers, the number of amino end groups doubles with each increase in generation, starting with generation zero (G(0)) PAMAM dendrimer that presents a theoretical maximum of four terminal amines to G10 that presents a theoretical maximum of 4096 end groups [140]. Glycodendrimers, which are generated by functionalizing the PAMAM scaffold with carbohydrates, have been employed for the study of a variety of biological processes mediated by protein–carbohydrate interactions [27]. By tethering multiple glycosides to the multivalent framework, PAMAM glycodendrimers can enhance the binding affinity of a generally weak monovalent interaction between a lectin and a carbohydrate to study biological processes involving protein carbohydrate interactions [146]. For example, glycodendrimers have been used to study multivalent interactions mediated by carbohydrate binding proteins known as lectins. Woller et al. used mannose functionalized dendrimer to control clustering of Concanavalin A (Con A), a plant lectin, in a generation-dependent and valency-dependent manner [147]. Binding of Con A was observed to alter the binding activity in hemagglutination and precipitation assays [147]. Con A binding to glycodendrimers was attenuated in a predictable manner by mixing carbohydrates that had lower and higher monovalent affinities on the dendrimer surface [148,149]. Goodman et al. used lactose functionalized dendrimers to study galectin-3 interactions, showing that aggregate formation was a function of the size of the multivalent framework and the number of nucleation sites [150]. Michel et al. then applied the lactose functionalized dendrimers to the study of galectin-3 mediated cellular aggregation, demonstrating inhibition of aggregation occurred when small glycodendrimers (i.e., G(2)) were used and larger glycodendrimers (i.e., G(6)) augmented aggregation [36].

**Figure 10:**
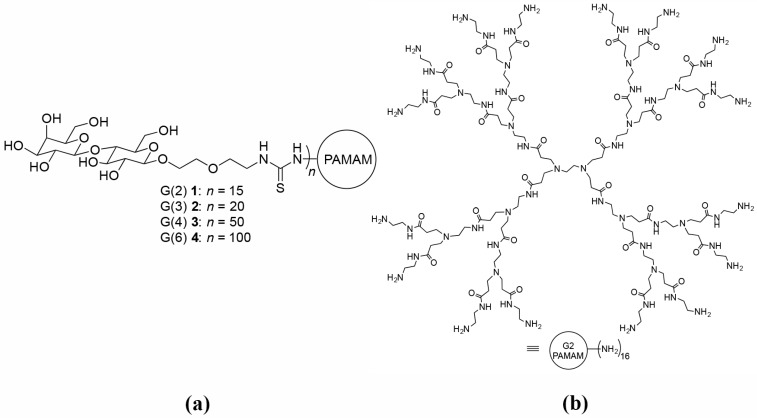
(**a**) Lactose functionalized poly(amidoamine) (PAMAM) dendrimers used; and (**b**) PAMAM framework.

Since presenting multiple glycosides on a multivalent framework enhances the strength and specificity of the interaction with another galectin [136,151], glycodendrimers **1**–**4** were also studied with galectin-1. Generations 2, 3, 4, and 6 PAMAM dendrimers were functionalized with lactose to generate the series of glycodendrimers. Synthesis and characterization were performed according to reported procedures [150]. Figure 10a shows the four generations of lactose-functionalized dendrimers used for these studies and the lactose loading of each generation as determined using nuclear magnetic resonance (NMR) and matrix assisted laser-desorption time of flight mass spectrometry (MALDI-ToF MS). The PAMAM framework is shown in Figure 10b.

To study the influence of synthetic multivalent ligands on galectin-1 mediated cellular processes, a variety of assays were performed. For example, the interaction of galectin-1 and surface adsorbed multivalent dendrimers **1**–**4** were investigated using an ELISA. The ELISA indicated that galectin-1 binds effectively to the glycodendrimers [152]. In addition, the binding of galectin-1 to glycodendrimers in solution was investigated using Dynamic Light Scattering (DLS) and Fluorescence Microscopy (FM) [89]. Glycodendrimers **1**–**4** organized galectin-1 into biologically active arrays that were applied to a cellular aggregation assay using DU145 prostate cancer cells (because they express Mucin 1, a cognate galectin-1 receptor [58,153]). The multivalent glycodendrimers altered the presentation of the galectin-1 to the cells, which inhibited homotypic aggregation.

## 6. Conclusions

Cancer is a leading cause of death worldwide. The malignant spread of cancer is advanced in part through multivalent protein carbohydrate interactions. Galectin-1, which is commonly over expressed in malignant cancer, mediates a variety of cellular processes in cancer progression by interacting with glycoconjugates in the tumor microenvironment. For example, galectin-1 has been found to arbitrate cellular aggregation, migration and invasion, tumor-induced angiogenesis, and T cell apoptosis. Studies using animal models support the critical role of galectin-1 in mediating tumor growth and metastasis. Taken together, these studies indicate that galectin-1 inhibition is a promising therapeutic strategy against cancer. 

Because galectin-1 mediates metastatic processes via multivalent interactions with glycoconjugates, traditional monovalent therapeutics are fundamentally ineffective. Synthetic, multivalent systems, which can simultaneously target multiple interactions, offer an auspicious alternative. In order to develop robust multivalent galectin-1 therapeutics, a better understanding of galectin-1 mechanisms in cancer is paramount. Here, materials designed to evaluate the impact of several features of the synthetic multivalent glycosystems on galectin-1 are presented. These systems are being used to probe the importance of flexibility and dynamics, linker length, functionalization adjacent to the carbohydrate, size of the framework, and number of carbohydrates on the binding interaction with galectin-1. Synthetic multivalent systems such as the ones described here are now providing important insights into the multivalent mechanisms of action of galectin-1. They are now being used to probe the mechanism of action of galectin-1 in cancer processes.

## Figures and Tables

**Table 1:** Galectin-1 binding partners and associated cancer processes.

Localization	Binding Partner	Biological Activities	Cell Type	References
Intracellular	H-Ras	H-Ras/MEK/ERK cascade activation	Bladder cancer	[78]
	Pro-24	β-catenin signaling inhibition	Colon cancer	[79]
	Gemin4	Pre-RNA splicing modulation	Cervical cancer	[80]
Extracellular	90K/Mac-2BP	Homotypic cell adhesion	Melanoma	[57]
	Mucin 1	Cell adhesion	Prostate cancer	[81]
	Laminin	Cell–ECM adhesion	Endothelial	[82]
	Fibronectin	Cell–ECM adhesion	Endothelial	[83]
	Neuropilin-1	Proliferation, migration, and adhesion induction	Endothelial	[84]
	VEGFR	Neovascularization activation	Endothelial	[9]
	CD45	Membrane redistribution, and T cell death induction	T cell	[85,86]
	CD43	Membrane redistribution, and T cell death induction	T cell	[10,85]
	CD7	T cell death induction	T cell	[85]

ECM, extracellular matrix; MEK, mitogen and extracellular signal regulated protein kinase; ERK, extracellular signal regulated protein kinase; VEGFR, vascular endothelial growth factor receptor.

## Abbreviations

Con A	Concanavalin A
CRD	Carbohydrate recognition domain
ECM	Extracellular matrix
MALDI-ToF	Matrix assisted laser-desorption time of flight
MMP	Matrix metalloproteinase
NMR	Nuclear Magnetic Resonance
PAMAM	Poly(amidoamine)
TF	Thomsen-Friedenreich
TF-PAA	Thomsen-Friedenreich polyacrylamide
VEGF	Vascular endothelial growth factor
VEGFR	Vascular endothelial growth factor receptor
